# Prognosis of liver abscess with no identified organism

**DOI:** 10.1186/s12879-019-4131-z

**Published:** 2019-05-31

**Authors:** Jai Hoon Yoon, Youn Jeong Kim, Sang Il Kim

**Affiliations:** 1 Division of Gastroenterology, Department of Internal Medicine, Hanyang University College of Medicine, Seoul, South Korea; 2 Department of Internal Medicine, College of Medicine, The Catholic University of Korea, Seoul, South Korea; 3 Division of Infectious Disease, Department of Internal Medicine, Incheon St. Mary’s Hospital, College of Medicine, The Catholic University of Korea, #56, Donsu-Ro, Bupyung-Gu, Incheon, South Korea; 4 Division of Infectious Disease, Department of Internal Medicine, Seoul St. Mary’s Hospital, College of Medicine, The Catholic University of Korea, Seoul, South Korea

**Keywords:** Liver abscess, Culture-negative, *E.coli*, *K.pneumoniae*

## Abstract

**Background:**

There are limited studies focusing on liver abscess with negative microbiological cultures. This study evaluated the clinical and prognostic differences of patients with culture-negative liver abscess (CNLA) compared to those with a positive culture (CPLA) and compared these factors between *K. pneumoniae* liver abscess (KLA) and *E. coli* liver abscess (ELA).

**Methods:**

A retrospective study of the patients who admitted with a liver abscess at two tertiary hospitals in Korea from 2012 to 2016 was performed.

**Results:**

Among a total of 402 patients with liver abscess, 61.2% had positive cultures. *K. pneumoniae* (*n* = 133) was the most common cause, followed by *E. coli* (*n* = 74). Patients with CPLA were significantly older (*p* = 0.02) and more frequently had cholelithiasis or biliary tract disease (*p* = 0.001) compared to patients with CNLA. In-hospital mortality (*p* = 0.63) and recurrence (*p* = 0.77) were no different between the two groups. The length of hospital stay was significantly longer in patients with CPLA (*p* = 0.03) compared with those with CNLA. Subgroup analysis for patients who received 3rd generation cephalosporins empirically showed that in-hospital mortality (*p* = 0.18) and recurrence (*p* = 0.27) were not also significantly different. Cholelithiasis, or biliary tract disease (*p* = 0.001), liver disease (*p* = 0.001), malignancy (*p* = 0.0001), and ESBL production (*p* = 0.0001) were found more frequently in patients with ELA compared with those with KLA.

**Conclusions:**

The prognosis of the CNLA patients was similar to that of the CPLA patients, although the length of hospital stay was shorter in the CNLA patients. The epidemiologic and clinical characteristics of the ELA patients are somewhat different than those of the KLA patients.

## Background

Pyogenic liver abscess is a common intraabdominal infection, and population–based studies in North America, the United States, and Taiwan have shown that the incidence of pyogenic liver abscess ranges from 2.3 to 17.59 per 100,000 person-years [1–3]. *E. coli* is known to be a predominant liver abscess pathogen; however, over the past three decades, the leading pathogen has begun shifting to *K. pneumoniae* worldwide [4–8]. Despite advances in microbiologic techniques, a causative pathogen is not identified in some cases of liver abscess. In one study, the sensitivity of the abscess culture was 90% for gram-positive cocci and 52% for gram–negative bacilli. However, the sensitivity was only 30% for gram positive-cocci and 39% for gram–negative bacilli in blood culture [9]. The best treatment for liver abscess is source control by drainage and administration of antibiotics that cover gram-negative bacilli, gram-positive cocci, and anaerobic bacteria [10, 11]. Empirically, 3rd-generation cephalosporins plus metronidazole or β-lactam/β-lactamase inhibitor are recommended initially, and antibiotics should later to be changed based on culture and antimicrobial susceptibility data. There are limited studies about liver abscess without a positive microbiological culture, and there are no guidelines for clinical practice in culture-negative liver abscess patients (CNLA). It remains unclear what kind of empiric antibiotics work better for CNLA patients. It is not clear whether the prognosis such as metastatic infection or mortality in CNLA patients is similar to that in patients with proven pathogens. This study was conducted to evaluate the clinical and prognostic differences in patients with CNLA compared to those with a positive microbiological culture, and to compare these factors between *K. pneumoniae* liver abscess (KLA) and *E. coli* liver abscess (ELA).

## Methods

### Study design

This retrospective study was conducted at Seoul St. Mary’s Hospital of Catholic University and the tertiary hospital of Hanyang University in Korea from January 2012 to December 2016.

### Patients and definitions

All hospitalized patients > 18 years old who were admitted to these two hospitals who were diagnosed with a liver abscess at discharge were selected from the medical record database. Liver abscesses were diagnosed based on the clinical presentation, laboratory findings, and imaging studies such as computed tomography, ultrasonography, or magnetic resonance imaging. Microbiologic data were collected from at least 1 set of blood cultures obtained prior to starting antibiotics or from pus obtained through an invasive procedure. CNLA was diagnosed if no organism grew on the initial blood culture obtained prior to antibiotics or from pus obtained through an invasive procedure. Polymicrobial infection was defined as the presence of ≥2 pathogens cultured from blood or pus. Metastatic infection was defined if a patient with liver abscess developed extrahepatic manifestations such as endophthalmitis, central nervous system infections, lung abscesses, and skin or soft tissue infections. Invasive procedures included aspiration, percutaneous drainage, and surgical interventions.

### Data collection

The following data were collected: age, sex, underlying disease, laboratory findings, microbiologic data, treatment strategy, complications, and clinical outcomes. In-hospital mortality was used as the main outcome for assessing mortality in patients with a liver abscess.

### Microbiologic data

Both aerobic and anaerobic cultures were performed for the blood and pus samples. Species identification and antimicrobial susceptibility were tested using VITEK automated systems (bioMérieux Vitek, USA), and interpreted according to guidelines established by the Clinical and Laboratory Standards Institute according to the Clinical and Laboratory Standards Institute (CLSI) criteria. Phenotypic confirmation of ESBL detection was performed using the double-disk diffusion method in our clinical microbiology laboratories, as recommended by the CLSI.

### Ethical approval

The Institutional Review Board (IRB) of the Seoul St. Mary’s Hospital (KC18REDI0751) and Hanyang University (2018–08–017-001) approved this study. The IRB waived the requirement to obtain written informed consent from the patients.

### Statistical analysis

The independent sample *t*-test or Kruskal-Wallis test was used to analyze continuous variables, and the Chi-square test or Fisher’s exact test was used for categorical variables. Statistical analysis was performed using SPSS 13.0 (SPSS Inc., Chicago, IL, USA), and a *p*-value of < 0.05 was considered statistically significant.

## Results

### Comparison of patients with liver abscess with or without positive microbiological culture

#### Demographic characteristics

**Fig. 1:**
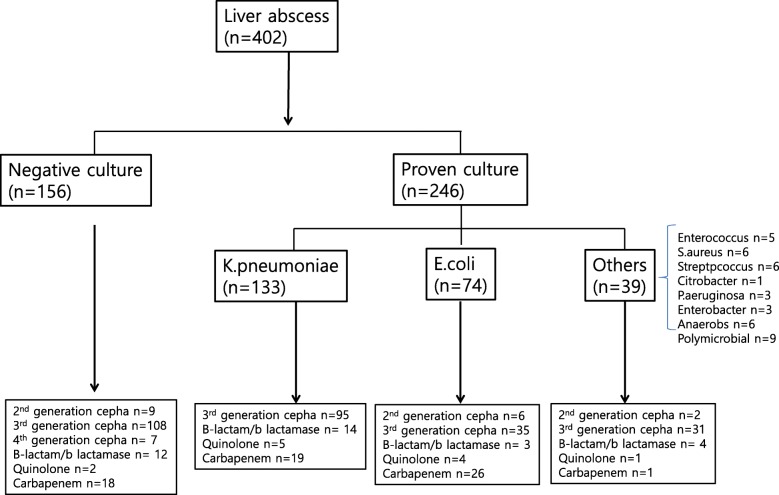
Flowchart of patient selection

Among a total of 402 patients with liver abscess, etiologic organisms were found in 61.2% (*n* = 246). Blood cultures were performed in 239 patients, and 41.4% (*n* = 99) were positive. Of 183 patients who underwent invasive procedures, 91.2% (*n* = 167) were found to harbor etiologic organisms through abscess culture, and among these patients, 113 had negative blood cultures. Of 246 culture-proven liver abscesses (CPLA), *K. pneumoniae* was the most common pathogen found (*n* = 133), followed by *E. coli* (*n* = 74) (Fig. 1). Table 1 shows the demographic characteristics of the patients with CPLA and CNLA. Those with CPLA were significantly older (median [IQR] 66 [8] vs. 61 [12] years old, *p* = 0.02) and more frequently had cholelithiasis or biliary tract disease (*n* = 35 (14.2%) vs. *n* = 6 (3.8%), *p* = 0.001) compared to patients with CNLA. Sex (*p* = 0.06); diabetes mellitus (*p* = 0.12), hypertension (*p* = 0.39), malignancy (*p* = 0.10) and transplant (*p* = 0.64) as a comorbidity; presentation with sepsis (*p* = 0.84); and ICU admission (*p* = 0.88) were no different between the two groups.

**Table 1:** Demographic characteristics of patients with liver abscess

	CNLA (*n* = 156)	CPLA (*n* = 246)	Causative organisms				*P* value
			*E.coli* (*n* = 74)	*K.pneumoniae* (*n* = 133)	*P* value*	Other (*n* = 39)	
Age, years, median (IQR)	61 (12)	66 (8)	66.5 (14.3)	66 (18)	0.95	66 (7)	0.02
Sex, male, n(%)	113 (72.4%)	156 (63.4%)	49 (66.2%)	79 (59.4%)	0.33	28 (71.8%)	0.06
Comorbidity							
Diabetes mellitus	35 (22.4%)	74 (30.1%)	24 (33.8%)	35 (26.3%)	0.26	14 (35.9%)	0.12
Hypertension	63 (40.4%)	89 (36.2%)	26 (35.1%)	48 (36.1%)	0.89	15 (38.5%)	0.39
Cholelithiasis and biliary tract disease	6 (3.8%)	35 (14.2%)	18 (24.3%)	11 (8.3%)	0.001	6 (15.4%)	0.001
Liver disease	12 (7.7%)	33 (13.4%)	18 (24.3%)	10 (7.5%)	0.001	5 (12.8%)	0.07
Chronic kidney disease	3 (1.9%)	5 (2.0%)	1 (1.4%)	3 (2.3%)	0.65	1 (2.6%)	0.94
Malignancy	24 (15.4%)	54 (22.0%)	27 (36.5%)	11 (8.3%)	0.0001	16 (41.0%)	0.10
Transplant	2 (1.3%)	2 (0.8%)	1 (1.4%)	1 (0.8%)	0.67	0 (0%)	0.64
Presentation as a sepsis	5 (3.2%)	7 (2.8%)	1 (1.4%)	6 (4.5%)	0.23	0 (0%)	0.84
ICU admission	22 (14.1%)	36 (14.6%)	11 (14.9%)	20 (15.0%)	0.97	5 (12.8%)	0.88
Bacteremia	–	99 (40.2%)	21 (28.4%)	56 (42.1%)	0.05	22 (56.4%)	–
ESBL production	–		23 (31.1%)	3 (2.3%)	0.0001		

*P value was calculated on two groups of *E.coli* liver abscess and *K.pneumoniae* liver abscess

#### Laboratory findings

Table 2 shows a comparison of the laboratory data between the two groups. There were no significant differences in the white blood cell count (*p* = 0.69), AST (*p* = 0.70), ALT (*p* = 0.53), alkaline phosphatase (*p* = 0.26), r-GTP (*p* = 0.10), total bilirubin (*p* = 0.26), or creatinine (*p* = 0.06). The platelet count was significantly lower (median 167 vs. 212, *p* = 0.001), and the CRP (median [IQR] 16.9 [8.7] vs. 13.3 [8.9], *p* = 0.01) and albumin (median[IQR] 3.1[0.5] vs. 3.0[0.4], *p* = 0.01) were higher in the CPLA patients compared with the CNLA patients.

**Table 2:** Laboratory findings of patients with liver abscess

	CNLA (*n* = 156)	CPLA (*n* = 246)	Causative organism				*P* value
			*E.coli* (*n* = 74)	*K.pneumoniae* (*n* = 133)	*P* value*	Other (*n* = 39)	
White blood cells, per mm^3^, median (IQR)	11,500 (5142)	12,095 (4692.5)	11,200 (8225)	13,130 (8560)	0.30	10,300 (5450)	0.69
Platelets(×10^3^), per mm^3^, median (IQR)	212 (102)	167 (104.5)	166 (136)	157 (126)	0.37	549 (93)	0.001
C-reactive protein, mg/dℓ, median (IQR)	13.3 (8.9)	16.9 (8.7)	14.1 (11.7)	20.7 (13.3)	0.61	11.3 (5.09)	0.01
AST, IU/L, median (IQR)	53.5 (48)	59 (35)	45 (51)	65.5 (60.3)	0.23	56 (73)	0.70
ALT, IU/L, median (IQR)	53 (50.5)	61 (37)	42 (48)	74.5 (72.6)	0.40	50 (34)	0.53
Alkaline phosphotase, IU/L, median (IQR)	139 (78)	132.5 (97.5)	196 (235)	121 (104.5)	0.0001	144 (91.5)	0.26
r-GTP, IU/L, median (IQR)	140 (126)	104 (112.7)	100 (263.7)	113 (143)	0.0007	94 (153)	0.10
Total bilirubin, mg/dℓ, median (IQR)	0.71 (0.8)	1.08 (0.94)	0.98 (1.58)	1.10 (1.18)	0.25	0.96 (0.99)	0.26
Albumin, g/dℓ, median (IQR)	3.0 (0.4)	3.1 (0.5)	3.1 (0.8)	3.2 (0.8)	0.33	3.1 (0.6)	0.01
Creatinine, mg/dℓ median (IQR)	0.81 (0.25)	0.92 (0.31)	0.85 (0.43)	1.01 (0.46)	0.94	0.85 (0.25)	0.06

*P value was calculated on two groups of *E.coli* liver abscess and *K.pneumoniae* liver abscess

#### Treatment and clinical outcomes

Invasive procedures, such as percutaneous drainage or surgery, were performed in 79.3% (*n* = 195) of CPLA patients and 69.2% (*n* = 108) of CNLA patients, and these differences were significant (*P* = 0.02) (Table 3). Metastatic infection developed more frequently in patients with CPLA (*n* = 12, 4.9%) compared to those with CNLA (*n* = 2, 1.3%); however, this difference was not significant (*p* = 0.05) (Table 3). Empirically, the most commonly used antibiotics were 3rd generation cephalosporins in all groups, and these were more frequently prescribed in patients with CNLA (*n* = 108, 69.2%) than in those with CPLA (*n* = 161, 65.4%) (Fig. 1). In-hospital mortality (*p* = 0.63) and recurrence (*p* = 0.77) were no different between the two groups. The length of hospital stay was significantly longer in CPLA patients (median [IQR] 17.9 [8] vs. 14.8 [7.75], *p* = 0.03) compared with CNLA patients.

**Table 3:** Treatment and clinical outcomes

	CNLA (*n* = 156)	CPLA (*n* = 246)	Causative organism				*P* value
			E.coli (*n* = 74)	K.pneumoniae (*n* = 133)	*P* value*	Other (*n* = 39)	
Invasive procedure, n(%)	108 (69.2%)	195 (79.3%)	54 (73.0%)	108 (81.2%)	0.17	33 (84.6%)	0.02
Metastatic infection, n(%)	2 (1.3%)	12 (4.9%)	4 (5.4%)	7 (5.3%)	0.96	1 (2.6%)	0.05
In-hospital mortality, n(%)	6 (3.8%)	12 (4.9%)	5 (6.8%)	4 (3.0%)	0.20	3 (7.7%)	0.63
Length of hospital stay, days, median (IQR)	14.8 (7.75)	17.9 (8)	20 (15)	18 (9.5)	0.09	17 (16)	0.03
Recurrence, n (%)	12 (7.7%)	17 (6.9%)	6 (8.1%)	5 (3.8%)	0.18	6 (15.4%)	0.77

*P value was calculated on two groups of E.coli liver abscess and K.pneumoniae liver abscess

#### Subgroup analysis for patients who received 3rd generation cephalosporins empirically

In-hospital mortality (CPLA 5.0% [8/161] vs. CNLA 1.9% [2/108], *p* = 0.18), recurrence (CPLA 5.0% [8/161] vs. CNLA 8.3% [9/108], *p* = 0.27) and metastatic infection (CPLA 5.0% [8/161] vs. CNLA 1.9% [2/108], *p* = 0.18) were not different between the two groups. The length of hospital stay was not different (CPLA median[IQR] 16 [11] days vs. CNLA median[IQR] 14.5 [10] days, *p* = 0.11).

### Comparison of liver abscess patients with *Klebsiella* and *E.coli*

#### Demographic characteristics

Table 1 shows a comparison between the demographic characteristics of patients with KLA (*n* = 133) and ELA (*n* = 74). Age (*p* = 0.95), diabetes mellitus (*p* = 0.26), hypertension (*p* = 0.89), and chronic kidney disease (*p* = 0.65) as underlying diseases were not significantly different between the 2 groups. However, cholelithiasis, or biliary tract disease, was identified more often in patients with ELA (*n* = 18, 24.3%) compared to those with KLA (*n* = 11, 8.3%) (*p* = 0.001). Liver disease (ELA *n* = 18 (24.3%) vs. KLA *n* = 10 (7.5%), *p* = 0.001) and malignancy (ELA *n* = 27 (36.5%) vs. KLA n = 11 (8.3%), *p* = 0.0001) were also found most frequently in patients with ELA. Patients with KLA had more bacteremia, although this difference was not significant (ELA *n* = 21 (31.1%) vs. KLA *n* = 56 (42.1%), *p* = 0.05). ESBL-producing organisms were observed more frequently in patients with ELA compared with those with KLA (ELA *n* = 23 (31.1%) vs KLA *n* = 3 (2.3%), *p* = 0.0001).

#### Laboratory findings

There were no significant differences in the white blood cell count (*p* = 0.30), platelet count (*p* = 0.37), C-reactive protein (*p* = 0.61), AST (*p* = 0.23) or ALT (*p* = 0.40). Alkaline phosphatase (*p* = 0.26) was significantly higher (median [IQR] 196 [235] vs. 121 [104.5], *p* = 0.0001), and the r-GTP (median [IQR] 100 [263.7] vs. 113 [143]. *p* = 0.0007) was lower in the ELA patients compared with the KLA patients (Table 2).

#### Treatment and clinical outcomes

In both the ELA and KLA patients, 3rd generation cephalosporins were the most frequently used empiric antibiotics, followed by carbapenems (Table 3). Invasive procedures were performed more often in patients with KLA (*n* = 108, 81.2%) than in those with ELA (*n* = 54, 73.0%); however, this difference was not significant (*p* = 0.17). There was also no difference between the 2 groups in metastatic infections (*p* = 0.96), in-hospital mortality (*p* = 0.20) or recurrence (*p* = 0.18). The length of hospital stay was longer in ELA patients, although this was not significantly different (median [IQ] 20 [13] vs. 18 [9.5] days, *p* = 0.09).

## Discussion

The epidemiologic profile of liver abscess differs between countries. *E. coli* was a prevalent cause of liver abscess until the mid-1980s; however, since then, *K. pneumoniae* has become increasingly important as a liver abscess pathogen [5, 12–16]. In our previous study, *Klebsiella pneumoniae* was also emerging as the main cause of liver abscess in our countries, followed by *E. coli* [17]. There have been reports of *Klebsiella* species associated with invasive syndromes showing extrahepatic complications in southeast Asian countries. However, there are few data on the clinical characteristics and prognosis for liver abscess with no etiologic organism identified or due to other pathogens.

There was no difference in the in-hospital mortality, metastatic infection or recurrence between patients with CNLA and CPLA. For patients with a liver abscess, empiric antibiotic coverage for gram-negative bacilli, gram-positive cocci, and anaerobes is recommended, and 3rd generation cephalosporins and/or metronidazole or β-lactam/β-lactamase inhibitor are the mainstay of treatment for liver abscess [10, 18–20]. After pathogen identification, antibiotics should be modified based on microbiologic data and in vitro susceptibility. In clinical practice, physicians have some difficulty selecting antibiotics, especially for liver abscess due to unknown organisms. Clinicians may feel tempted to use broader, newer, and more expensive antibiotics. In our study, 3rd generation cephalosporins were the most frequently administered antibiotics in all patients with liver abscess. Subgroup analysis including patients with liver abscess who received 3rd generation cephalosporins showed that there was no difference in the in-hospital mortality and recurrence in CNLA and CPLA patients. It is acceptable that antibiotic selection for CNLA was similar to that for KLA because most patients with KLA, especially in cases of metastatic infection, had community-acquired infections, and the percentage of antibiotic resistant cases of *Klebsiella pneumoniae* was extremely low [15, 21]. However, host factors and local epidemiologic data must also be considered for antibiotic selection.

Our study demonstrated culture positivity among liver abscess patients in 41.4% by blood culture and 91.2% by invasive procedure. A population-based study in the United States found that 53.4% of patients had an identifiable organism on blood culture [1]. This finding suggests that advances in diagnostic methods have enhanced organism identification, and applications in real clinical situations are needed.

In our study, there were some differences in demographic characteristics between the ELA and KLA patients. ELA was associated with liver disease, biliary tract disease or malignancy, similar to previous results [13, 22, 23]. The percentage with ESBL production was higher in ELA than in KLA. In patients with ELA, underlying disease is is more likely to be associated with exposure to antibiotics or hospital admission, which may be associated with antibiotic resistance. Clinicians should consider broader spectrum antibiotics for patients with liver abscess if the patients have a malignancy or underlying biliary disease, as this may be suggestive of hospital acquired infection.

In our study, metastatic infection was also seen in patients with ELA, although there are several studies showing that the extrahepatic manifestation of liver abscess is typically a devastating complication in hypervirulent *K. pneumoniae* [12, 14, 17]. Further studies on virulence factors associated with invasive syndromes in *E. coli* are also needed.

Pyogenic liver abscess is a potentially life–threatening infection with high mortality. However, our study showed a relatively low in-hospital mortality of 5.7% compared to previous studies. [4, 13, 24, 25] In several studies, the mortality was associated with age, comorbidities, and prompt surgical modality including radiologic intervention [1, 26, 27]. We did not evaluate any factors associated with mortality in this study. However, the high percentage of invasive procedures performed in 75% of patients with liver abscess may be associated with favorable outcomes.

Our study has some limitations. It is a retrospective study that included only 2 hospitals. We did not examine factors related to organism identification; for example, the administration of antibiotics prior to culture collection might be associated with a false negative culture result.

## Conclusion

Our study suggests that the prognosis including mortality, recurrence and metastatic infection was no different between the patients with CNLA and CPLA, although the length of hospital stay was shorter for CNLA patients compared with CPLA patients. The epidemiologic and clinical characteristics of the ELA patients are different from those of the KLA patients.

## Data Availability

The data analyzed during this study are included in this paper. Some of the datasets are available from the corresponding author upon reasonable request.

## Abbreviations

CNLA: Culture-negative liver abscess
CPLA: Culture-positive liver abscess
ELA: *E.coli* liver abscess
KLA: *K.pneumoniae* liver abscess
